# Distinct neuroinflammatory signatures exist across genetic and sporadic amyotrophic lateral sclerosis cohorts

**DOI:** 10.1093/brain/awad243

**Published:** 2023-07-14

**Authors:** Olivia M Rifai, Judi O’Shaughnessy, Owen R Dando, Alison F Munro, Michael D E Sewell, Sharon Abrahams, Fergal M Waldron, Christopher R Sibley, Jenna M Gregory

**Affiliations:** Translational Neuroscience PhD Programme, Centre for Clinical Brain Sciences, University of Edinburgh, Edinburgh, EH8 9XD, UK; Centre for Clinical Brain Sciences, University of Edinburgh, Edinburgh, EH16 4SB, UK; UK Dementia Research Institute, University of Edinburgh, Edinburgh, EH16 4SB, UK; Euan MacDonald Centre for Motor Neurone Disease Research, University of Edinburgh, Edinburgh, EH16 4SB, UK; Centre for Discovery Brain Sciences, University of Edinburgh, Edinburgh, EH8 9XD, UK; Centre for Clinical Brain Sciences, University of Edinburgh, Edinburgh, EH16 4SB, UK; Euan MacDonald Centre for Motor Neurone Disease Research, University of Edinburgh, Edinburgh, EH16 4SB, UK; UK Dementia Research Institute, University of Edinburgh, Edinburgh, EH16 4SB, UK; Centre for Discovery Brain Sciences, University of Edinburgh, Edinburgh, EH8 9XD, UK; Simons Initiative for the Developing Brain, University of Edinburgh, Edinburgh, EH8 9XF, UK; Cancer Research UK Edinburgh Centre, Institute of Genetics and Cancer, The University of Edinburgh, Edinburgh, EH4 2XU, UK; Translational Neuroscience PhD Programme, Centre for Clinical Brain Sciences, University of Edinburgh, Edinburgh, EH8 9XD, UK; UK Dementia Research Institute, University of Edinburgh, Edinburgh, EH16 4SB, UK; Human Cognitive Neuroscience-Psychology, School of Philosophy, Psychology and Language Sciences, University of Edinburgh, Edinburgh, EH8 9AD, UK; Institute of Medical Sciences, University of Aberdeen, Aberdeen, AB25 2ZD, UK; Euan MacDonald Centre for Motor Neurone Disease Research, University of Edinburgh, Edinburgh, EH16 4SB, UK; Centre for Discovery Brain Sciences, University of Edinburgh, Edinburgh, EH8 9XD, UK; Simons Initiative for the Developing Brain, University of Edinburgh, Edinburgh, EH8 9XF, UK; Institute of Quantitative Biology, Biochemistry and Biotechnology, School of Biological Sciences, University of Edinburgh, The King’s Buildings, Edinburgh, EH9 3FF, UK; Institute of Medical Sciences, University of Aberdeen, Aberdeen, AB25 2ZD, UK

**Keywords:** amyotrophic lateral sclerosis, frontotemporal dementia, C9orf72, neuroinflammation, cognitive impairment, post-mortem tissue

## Abstract

Amyotrophic lateral sclerosis (ALS) is a neurodegenerative disease characterized by progressive loss of upper and lower motor neurons. ALS is on a pathogenetic disease spectrum with frontotemporal dementia, referred to as ALS-frontotemporal spectrum disorder (ALS-FTSD). For mutations associated with ALS-FTSD, such as the *C9orf72* hexanucleotide repeat expansion, the molecular factors associated with heterogeneity along this spectrum require further characterization.

Here, using a targeted NanoString molecular barcoding approach, we interrogate neuroinflammatory dysregulation and heterogeneity at the level of gene expression in post-mortem motor cortex tissue from a cohort of clinically heterogeneous C9-ALS-FTSD cases.

We identified 20 dysregulated genes in C9-ALS-FTSD, with enrichment of microglial and inflammatory response gene sets. Two genes with significant correlations to available clinical metrics were selected for validation: *FKBP5*, a correlate of cognitive function, and brain-derived neurotrophic factor (*BDNF*), a correlate of disease duration. FKBP5 and its signalling partner, NF-κB, appeared to have a cell type-specific staining distribution, with activated (i.e. nuclear) NF-κB immunoreactivity in C9-ALS-FTSD. Expression of *BDNF*, a correlate of disease duration, was confirmed to be higher in individuals with long compared to short disease duration using BaseScope™ *in situ* hybridization. Our analyses also revealed two distinct neuroinflammatory panel signatures (NPS), NPS1 and NPS2, delineated by the direction of expression of proinflammatory, axonal transport and synaptic signalling pathways. We compared NPS between C9-ALS-FTSD cases and those from sporadic ALS and SOD1-ALS cohorts and identified NPS1 and NPS2 across all cohorts. Moreover, a subset of NPS was also able to separate publicly available RNA sequencing data from independent C9-ALS and sporadic ALS cohorts into two inflammatory subgroups.

Importantly, NPS subgroups did not clearly segregate with available demographic, genetic, clinical or pathological features, highlighting the value of molecular stratification in clinical trials for inflammatory subgroup identification. Our findings thus underscore the importance of tailoring therapeutic approaches based on distinct molecular signatures that exist between and within ALS-FTSD cohorts.

## Teaser

Rifai *et al.* characterize neuroinflammation in brain tissue from patients with ALS and identify two distinct inflammatory signatures, which do not appear to segregate with genetics or clinical symptoms. These signatures may be important for the stratification of clinical trials for appropriate outcome measurement.

## Introduction

Hexanucleotide repeat expansions (HREs) in the *C9orf72* gene are one of the most common mutations associated with amyotrophic lateral sclerosis-frontotemporal spectrum disorder (ALS-FTSD).^1-4^ Clinical manifestations of disease associated with *C9orf72* HRE are variable; presentations can involve motor or cognitive symptoms related to ALS-FTSD, or other symptoms such as parkinsonism and psychosis.^5-8^ This heterogeneity occurs despite a seemingly unifying neuropathological phenotype characterized by p62, TDP-43 and dipeptide repeat protein (DPR) deposits.^9-14^ Clinical heterogeneity has the potential to be a large confounding factor in clinical trials including people with ALS-FTSD, which often employ outcome measures based on clinical phenotypes. Thus, a better understanding of heterogeneity in C9-ALS-FTSD and in people with ALS-FTSD generally, and whether molecular heterogeneity maps onto clinical heterogeneity, is critical for informing the design of therapeutics intended to reduce specific symptom burden, as well as for improved trial stratification, so that endpoints can be more meaningfully measured.

One potential factor contributing to heterogeneity in people with ALS-FTSD is immune function and its related inflammatory processes. Inflammatory mediators such as regulatory T cells and interleukins have previously been shown to be associated with the rate of disease progression.^15-17^ Furthermore, differences in neuroinflammatory markers like CHIT1 and GFAP have been observed in the CSF between ALS and frontotemporal dementia (FTD) patients,^18,19^ suggesting that differential processes, particularly those regulated by neuroglia, may be occurring between conditions.^18^ As *C9orf72* is highly expressed in microglia,^20^ the resident immune cells of the CNS, it has been suggested that microglia may be particularly susceptible to any negative consequences of a change in normal C9orf72 protein function, thus triggering immune dysfunction,^21^ as evidenced by knockout *C9orf72* models.^22,23^ We have previously shown with immunohistochemical staining of post-mortem tissue that microglial activation is elevated in the language-related region Brodmann area (BA) 39 in language-impaired C9-ALS-FTSD cases.^24^ Additionally, we have demonstrated with random forest modelling that microglial staining is an accurate classifier of C9-ALS-FTSD, with better sensitivity and specificity to disease than other markers such as astrocyte activation marker, GFAP, and phosphorylated TDP-43 aggregate marker, pTDP43.^24^ Thus, further characterization of inflammatory heterogeneity in C9-ALS-FTSD, especially at a molecular level, is warranted to understand how these pathways can be more specifically targeted to harness their therapeutic potential. To further investigate this, we examined a cohort of cases with *C9orf72* HRE that encompasses C9-ALS cases with varying degrees of cognitive dysfunction, including cases with normal cognition, single-domain cognitive dysfunction and FTD (Table 1). This cohort will herein be referred to as the C9-ALS-FTSD cohort.

**Table 1:** C9-ALS-FTSD cohort demographics

Case number	Sex	Age at death (y)	Clinical diagnoses	Disease duration (months)	Region of onset	ECAS	ALSFRS	Neuronal pTDP43 burden	Glial pTDP43 burden
C9-1	F	63	ALS	25	Lower limb	Yes (unimpaired)	No	2	2
C9 -2	M	50	ALS	29	Bulbar	Yes (language dysfunction)	Yes (4 data-points)	2	3
C9- 3	F	63	ALS	30	Upper limb	No	Yes (3 data-points)	2	2
C9-4	F	62	ALS	37	Mixed	Yes (unimpaired)	Yes (6 data-points)	1	2
C9-5	F	65	ALS	44	Lower limb	No	Yes (2 data-points)	1	2
C9-6	M	65	ALS	50	Upper limb	No	Yes (1 data-point)	1	1
C9-7	M	62	ALS	50	Lower limb	Yes (executive dysfunction)	Yes (2 data-points)	2	0
C9-8	M	43	ALS	57	Bulbar	Yes (language dysfunction)	No	1	2
C9-9	M	58	ALS	87	Lower limb	Yes (language dysfunction)	Yes (3 data-points)	3	3
C9-10	F	63	ALS, FTD, schizophrenia	119	Upper limb	Yes (FTD, 3 domains)	No	2	3
Control 1	F	65	Hypertension, cardiomyopathy	N/A	N/A	N/A	N/A	0	0
Control 2	M	50	None	N/A	N/A	N/A	N/A	1	0
Control 3	F	57	None	N/A	N/A	N/A	N/A	0	0
Control 4	F	57	None	N/A	N/A	N/A	N/A	0	0
Control 5	F	71	Depression, paranoid schizophrenia	N/A	N/A	N/A	N/A	0	0
Control 6	M	58	Depression	N/A	N/A	N/A	N/A	1	0
Control 7	F	59	Hypertension	N/A	N/A	N/A	N/A	0	0
Control 8	M	44	None	N/A	N/A	N/A	N/A	0	0
Control 9	M	63	None	N/A	N/A	N/A	N/A	0	0
Control 10	F	61	Depression, MS, hypothyroidism	N/A	N/A	N/A	N/A	0	0

ALS = amyotrophic lateral sclerosis; ALSFRS = ALS functional rating scale; ECAS = Edinburgh Cognitive and Behavioural ALS Screen; F = female; FTD = frontotemporal dementia; M = male; MS = multiple sclerosis; N/A = not applicable; y = years.

The information in this cohort has also been published in a previous study investigating the same cohort.^24^

To date, few studies have taken a targeted approach to measuring the expression of neuroinflammatory genes in a C9-ALS-FTSD cohort, particularly in post-mortem tissue. One recent study observed a general enriched immune response in post-mortem frontal cortex tissue from *C9orf72* HRE carriers,^25^ though this response was not explored further as the focus of the study. To interrogate inflammatory dysregulation in this context at a molecular level, we performed NanoString molecular barcoding on deeply clinically phenotyped post-mortem motor cortex from our C9-ALS-FTSD cohort (Table 1) to explore differential expression of 770 genes in an nCounter neuroinflammation panel. We identified 20 significantly differentially expressed genes in C9-ALS-FTSD, with clustering of therapeutically relevant gene expression patterns. We compared gene expression patterns with immunohistochemical data from our previous study to examine relationships between gene dysregulation and neuropathological staining.^24^ We also performed regional validation of two genes correlating with clinical scores using both immunohistochemical and BaseScope™ *in situ* hybridization techniques. Finally, we identified two distinct molecular signatures across C9-ALS-FTSD (*n* = 10), sporadic ALS (sALS) (*n* = 18) and SOD1-ALS (*n* = 5) cohorts, as well as in publicly available frontal cortex RNA sequencing data from independent C9-ALS and sALS cohorts.^26^

## Materials and methods

### Case identification and cognitive profiling

Post-mortem tissue from cases with ALS-FTSD (*n* = 33) was obtained from the Medical Research Council (MRC) Edinburgh Brain Bank (Tables 1 and 2). For genetic classification of all ALS cases, repeat-primed polymerase chain reaction (PCR) was carried out for *C9orf72* HRE identification and whole genome sequencing was carried out to identify other ALS-associated mutations. SOD1-ALS cases were confirmed to have an I114T missense mutation.^27^ Sporadic cases had no family history of ALS and no ALS-associated mutations identified through gene panel analysis.^27^ Post-mortem tissue from controls that were age- and sex-matched to C9-ALS-FTSD cases (*n* = 10) and had no history of neurological conditions or neurodegenerative pathology were obtained from the Edinburgh Sudden Death Brain Bank. Post-mortem tissue was collected with ethics approval from East of Scotland Research Ethics Service (16/ES/0084) in line with the Human Tissue (Scotland) Act (2006); the use of post-mortem tissue for studies was approved by the Edinburgh Brain Bank ethics committee and the Academic and Clinical Central Office for Research and Development (ACCORD) medical research ethics committee (AMREC). Clinical data were collected for the Scottish Motor Neurone Disease Register (SMNDR) and Care Audit Research and Evaluation for Motor Neurone Disease (CARE-MND) platform,^28^ with ethics approval from Scotland A Research Ethics Committee (10/MRE00/78 and 15/SS/0216). Donor patients underwent neuropsychological testing with the Edinburgh Cognitive and Behavioural ALS Screen (ECAS).^29^ Clinical correlates of motor dysfunction/disease progression include disease duration (months) and sequential ALS functional rating scale (ALSFRS) data-points. Clinical correlates of cognition include ECAS scores for ALS-specific and ALS non-specific subdomain scores. All patients consented to use of their data during life.

**Table 2:** Sporadic ALS and SOD1-ALS cohort demographics

Case number	Sex	Age at death (y)	Clinical diagnoses	Disease duration (months)	ECAS	Neuronal pTDP43 burden	Glial pTDP43 burden
sALS 1	F	78	ALS	11	No	1	1
sALS 2	F	76	ALS	12	No	2	2
sALS 3	M	90	ALS	14	No	0	1
sALS 4	M	70	ALS	16	Yes (unimpaired)	1	2
sALS 5	M	57	ALS, FTD	20	Yes (FTD, 3 domains)	1	1
sALS 6	F	68	ALS	24	Yes (behavioural dysfunction)	1	1
sALS 7	M	73	ALS	24	Yes (executive dysfunction)	0	0
sALS 8	F	61	ALS	24	No	2	0
sALS 9	M	75	ALS	50	No	1	2
sALS 10	M	71	ALS	52	No	1	1
sALS 11	F	50	ALS	54	Yes (unimpaired)	1	1
sALS 12	F	72	ALS	55	Yes (unimpaired)	3	3
sALS 13	F	72	ALS	60	No	2	1
sALS 14	M	61	ALS	94	No	2	2
sALS 15	F	81	ALS	98	No	2	1
sALS 16	F	66	ALS	99	Yes (fluency dysfunction)	0	2
sALS 17	F	76	ALS, FTD	130	Yes (FTD, 3 domains)	1	1
sALS 18	M	66	ALS	134	Yes (unimpaired)	1	1
SOD1-ALS 1	M	46	ALS	14	Yes (unimpaired)	0	0
SOD1-ALS 2	M	71	ALS	38	No	0	0
SOD1-ALS 3	M	64	ALS	67	Yes (unimpaired)	0	0
SOD1-ALS 4	F	59	ALS	98	Yes (unimpaired)	0	0
SOD1-ALS 5	F	75	ALS	127	No	0	0

ALS = amyotrophic lateral sclerosis; ECAS = Edinburgh Cognitive and Behavioural ALS Screen; F = female; FTD = frontotemporal dementia; M = male; sALS = sporadic ALS; y = years.

### NanoString sequencing and analysis

RNA from human tissue was extracted using the RNAstorm FFPE RNA extraction kit (Cell Data Sciences) on two 10 µm curls per sample cut from BA4. RNA was eluted in 50 μl nuclease-free water, after which sample concentrations were measured using a NanoDrop 1000 (ThermoFisher Scientific). Samples that did not meet the minimum 60 ng/μl were concentrated using an Eppendorf Concentrator Plus (Eppendorf) for 10 min at 45°C, measured again, and concentrated for an additional 5 min at 45°C if necessary. Samples were diluted in nuclease-free water to a final concentration of 600 ng RNA in 10 μl water for NanoString sequencing. Sequencing was performed by Host and Tumour Profiling Unit (HTPU) Microarray Services with the nCounter neuroinflammation panel (for more information see https://nanostring.com/products/ncounter-assays-panels/neuroscience/neuroinflammation/), which includes 770 genes, expressed by various cell types, and related to immunity and inflammation, neurobiology and neuropathology, and metabolism and stress^30^ (Supplementary material). All samples passed routine quality control checks. Differential gene expression analyses between control and ALS cohorts were performed in RStudio (R version 4.1.1)^31^ using ‘DESeq2’^32^ (version 1.32.0) with ‘RUVSeq’^33^ (version 1.26.0) to estimate and regress out unwanted variation (*k =* 3 factors of unwanted variance^34^). *P*-values were adjusted using a Benjamini-Hochberg false discovery rate (FDR) threshold (*P <* 0.05). Plots were made using the ‘ggplot2’ package^35^ (version 3.3.5) in R. Gene ontology (GO) enrichment analysis^36^ was performed with the ‘topGO’ package (version 2.44.0)^37^ in R, and gene set analysis correlation adjusted mean rank (CAMERA)^38^ from the ‘limma’ package^39^ (version 3.48.3) in R was performed using the Molecular Signatures Database (MSigDb)^40,41^ GO category terms, with the nCounter neuroinflammation gene panel as the background gene set and gene annotations taken from Ensembl (version 96).^42^ GO and CAMERA results were considered significant if −log_10_(*P*-value) > 1.3; significant CAMERA results were filtered for gene sets with *n =* 10+ genes. Clustering analyses were performed on housekeeping-normalized and scaled (*z*-transformed) counts using the ‘pheatmap’ package^43^ (version 1.0.12) in R. Correlations of immunohistochemistry (IHC) and ECAS data with housekeeping-normalized counts were calculated with the ‘corrplot’ package^44^ (version 0.91) in R, using Spearman’s test. Gene set testing was also performed with cell type-specific gene sets derived from published Brain RNA-Seq data^20^ to determine cell type-specific dysregulation of transcripts. For this analysis, ratios were calculated for the expression of each gene in each cell type compared to its maximum expression in any other cell type. ‘Human_(cell type)_5_times’ indicates all genes for which the calculated ratio is >5, ‘human_(cell type)_10_times’ indicates all genes for which the calculated ratio is >10, and ‘human_(cell type)_top100’ indicates the 100 genes with the highest ratio for that cell type, that is, the most specific genes for each cell type. Differential expression analysis between neuroinflammatory panel signature (NPS1 and NPS2) cases in the C9-ALS-FTSD cohort was conducted as described earlier, and all genes with an unadjusted *P*-value < 0.05 and adjusted *P*-value ≠ NA (i.e. not available due to low mean normalized counts) were taken through clustering analyses with the ‘pheatmap’ package using housekeeping-normalized and scaled (*z*-transformed) counts^43^ (version 1.0.12) in R.

### Public RNA sequencing data analysis

A raw count matrix of publicly available frontal cortex and cerebellum RNA sequencing data^26^ were accessed via the NCBI Gene Expression Omnibus (accession number GSE67196). Data were divided by brain region and counts for C9-ALS and sALS cases were extracted. Data were variance stabilized and scaled (i.e. *z*-transformed) across samples using ‘DESeq2’^32^ (version 1.32.0) in RStudio (R version 4.1.1).^31^ Clustering analyses were performed for each region using the ‘pheatmap’ package^43^ (version 1.0.12) in R. Heat maps included equivalent demographic, clinical or pathological information available with the public data analysed: sex, region of onset and disease duration.

### Immunohistochemistry

Post-mortem brain tissue was obtained from BA4, BA39, BA44, BA46 and fixed in 10% formalin for a minimum of 72 h. These regions were selected for their associations with clinical phenotypic correlates as we have shown previously^29^: BA4, motor; BA39, language; BA44, fluency and language; and BA46, executive function. For the FKBP5/NF-κB validation dataset, an additional case was included due to differences in tissue availability at the time of request. Tissue was dehydrated in a 70–100% ascending alcohol series and subsequently washed three times for 4 h in xylene. Three 5 h paraffin wax embedding stages were performed, after which formalin-fixed, paraffin-embedded (FFPE) tissue was cooled and sectioned on a microtome (ThermoFisher Scientific) into 4 μm serial sections. Sections were placed on Superfrost (ThermoFisher Scientific) microscope slides and left to dry overnight at 40°C. Sections were dewaxed with successive xylene washes, hydrated with alcohol, and treated with picric acid to remove formalin pigment and quench lipofuscin. For NF-κB staining, antigen retrieval was carried out in Tris-EDTA buffer (pH 9) in a pressure cooker for 30 min, after which a Novolink Polymer detection system^45^ was used with an Abcam anti- NF-κB antibody (Abcam) at a 1 in 1500 dilution. For FKBP5 staining, antigen retrieval was carried out in citric acid buffer (pH 6) in a Pressure King Pro pressure cooker for a 20 min cycle; samples were heated to 140°C and incubated for 5 min, after which pressure was manually released. The Novolink Polymer detection system (Leica Biosystems) was then used with an anti-FKBP5 antibody (OriGene) at a 1 in 80 dilution. Staining was performed with 3,3′-diaminobenzidine (DAB) chromogen and counterstained with haematoxylin, as per standard operating procedures, after which slides were dehydrated, washed in xylene, and coverslips mounted using DPX mountant (Sigma Aldrich). For sequential staining, slides initially stained with NF-κB or FKBP5 were soaked in xylene overnight, after which the coverslips were carefully removed, and the slides were soaked for several more hours until the DPX mountant had dissolved off the sections. Slides were restained according to standard operating procedures mentioned above, from hydration, to citric acid antigen retrieval with a pressure cooker, to staining with an anti-Iba1 antibody (Abcam) at a 1 in 3000 dilution. Protocols for CD68, Iba1, pTDP43 and GFAP staining were described previously.^24^ Manual grading of neuronal and glial TDP-43 burden was performed by a pathologist (J.M.G.) using a scale from 0 to 3, as outlined in a previous study.^46^

### Image analysis

For analysis of NF-κB and FKBP5 immunohistochemical staining, whole tissue sections were scanned with Brightfield at ×40 magnification using a Hamamtsu NanoZoomer XR [Hamamatsu Photonics (UK) Ltd]. Using NDP.view2 viewing software (Hamamatsu), regions of interest (ROIs) were taken from key regions for quantification. Three ROIs were taken from grey matter regions including layer V neurons, and three ROIs were taken from white matter regions. ROIs were analysed with QuPath software^47^ cell segmentation; cells were segmented using a watershed method based on haematoxylin counterstaining, with different parameters for grey and white matter and for neurons and glia to best distinguish between cell types. Full scripts used for the automated cell segmentation and quantification of NF-κB and FKBP5 are included in the Supplementary material. Cells were classified as nuclear- and/or cytoplasmic-positive for each stain based on the DAB mean intensity of each compartment. Measurements were exported at the image (number of nuclear- and/or cytoplasmic-positive cells) and cell level (intensity and morphological features). Nuclear and cytoplasmic intensities were averaged across all cells and ROIs for each case to avoid pseudoreplication, and a nuclear/cytoplasmic ratio was calculated for each case. Data were visualized in RStudio with the ‘ggplot2’ package^35^ (version 3.3.5). Results were presented as ungrouped or grouped by brain region, grey or white matter, and vascular or non-vascular adjacent. Analysis methods for CD68, Iba1, pTDP43 and GFAP staining can be found in our previous study.^24^

### BaseScope™ *in situ* hybridization

*In situ* hybridization was performed on tissue sections using BaseScope™ reagents (Advanced Cell Diagnostics) as per the manufacturer’s instructions^48^ and as described previously.^49^ Probe hybridization was performed using BaseScope™ probes for *BDNF* mRNA transcripts. Slides were counterstained using haematoxylin and lithium carbonate, washed in xylene, and coverslips were mounted using DPX Mountant. For BDNF BaseScope™ *in situ* hybridization, due to the sparsity of mRNA transcripts, expression was quantified manually by a pathologist (J.M.G.), who was blinded to clinical data, and a second rater (O.M.R.), who performed a 20% validation check; no discrepancies were identified between raters. Expression was quantified using a product score composed of two factors to account for multiple aspects of abundance: total transcript count per high-power field (×40) × total number of transcript-positive cells per high-power field. Ten high-power fields were evaluated and averaged across each case.

### Statistical analyses

For DESeq2 differential expression analysis, a Benjamini-Hochberg FDR threshold (*P <* 0.05) was used to determine significance. For comparisons and correlations, normality of data distribution was evaluated with Shapiro-Wilk’s test. Datasets that were found to have a majority normal distribution were subjected to parametric tests (i.e. unpaired *t*-test for nuclear/cytoplasmic intensity ratio analysis) and datasets that were found to be majority non-normal were subjected to non-parametric tests (i.e. Spearman’s test for correlations between immunohistochemical and NanoString data, and BDNF abundance and disease duration).

## Results

### Two distinct neuroinflammatory signatures exist in C9-ALS-FTSD

**Figure 1:**
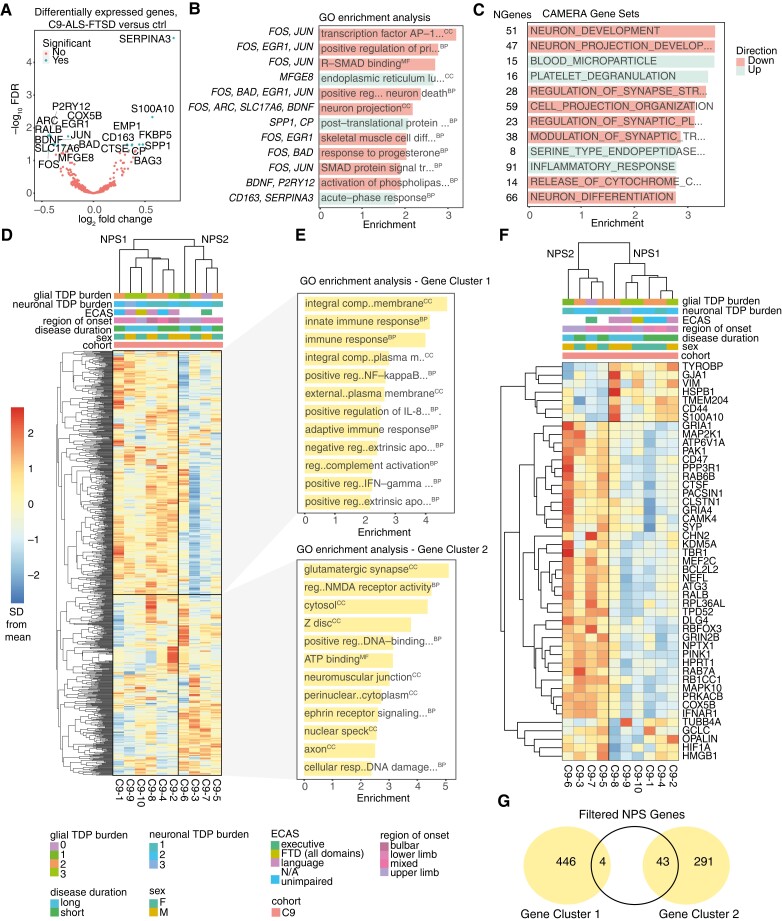
**Two distinct neuroinflammatory signatures exist in C9-ALS-FTSD.** (**A**) Volcano plot showing differentially expressed genes between C9-ALS-FTSD cases and controls by log_2_ fold change and −log_10_*P*-value. Non-significant genes are represented in red and significant genes [i.e. above the Benjamini-Hochberg false discovery rate (FDR) threshold of *P*-adjusted < 0.05] are represented in blue. (**B**) Gene ontology (GO) enrichment analysis of genes enriched in C9-ALS-FTSD cases by type with −log_10_(*P*-value) score showing the top 12 most differentially expressed gene sets. Italicized terms indicate downregulation; key genes for each term are shown to the *left*. (**C**) CAMERA gene set analysis of gene sets dysregulated in amyotrophic lateral sclerosis (ALS) cases, with the number of genes for each set shown to the *left*, showing the top 12 most differentially expressed gene sets. (**D**) Clustered neuroinflammation panel heat map including expression of entire NanoString panel (770 genes), showing two distinct neuroinflammatory panel signatures (NPS1 and NPS2) in C9-ALS-FTSD. Gene Clusters 1 and 2 are boxed, with opposite directions of expression between NPS. Clinical (region of onset, disease duration, ECAS) and pathological (neuronal and glial pTDP-43 burden) keys are shown. (**E**) GO enrichment analysis for Gene Clusters 1 and 2 (that define NPS1 and NPS2) showing top 12 most differentially expressed gene sets. Italicized terms indicate downregulation. (**F**) Clustered neuroinflammation panel heat map including only differentially expressed genes between C9-ALS-FTSD cases with NPS1 and NPS2, showing two distinct NPS, with genes listed on the right. (**G**) Venn diagram showing overlap between genes from the filtered NPS gene list in **E** with Gene Cluster 1 and 2 from **D** and **E**. BP = biological process; CC = cellular component; ECAS = Edinburgh Cognitive and Behavioural ALS Screen; F = female; FTD = frontotemporal dementia; M = male; MF = molecular factor; N/A = not applicable; SD = standard deviation.

To explore *C9orf72* mutation-related changes at the gene expression level, motor cortex (BA4) tissue was sequenced using NanoString molecular barcoding, which provides accurate mRNA counts without the need for amplification steps that favour highly abundant transcripts.^30^ This method circumvents RNA degradation issues related to post-mortem autolysis, as probes bind to the central, most preserved portion of mRNA transcripts. A ‘neuroinflammation panel’ of 770 genes expressed by different cell types and related to immunity and inflammation, neurobiology and neuropathology, and metabolism and stress was used.^30^ To first characterize disease-related dysregulation in the cohort, differential expression analysis between C9-ALS-FTSD (*n* = 10) and controls (*n* = 10) (Table 1) was conducted; raw and processed gene expression data are available in the Supplementary material. The analysis revealed a list of 20 genes that were significantly differentially expressed between C9-ALS-FTSD cases and controls (Fig. 1A). The microgliosis we observed previously in C9-ALS-FTSD^24^ is supported here by the upregulation of *CD163*, a marker of macrophage activity, and the downregulation of *P2RY12*, a marker of microglial homeostasis.^50,51^ The 20 significantly differentially expressed genes clustered into two similarly sized groups, those that were upregulated in C9-ALS-FTSD (*SERPINA3*, *S100A10*, *FKBP5*, *EMP1*, *CD163*, *SPP1*, *CP*, *CTSE*, *BAG3*) and those that were downregulated (*ARC*, *RALB*, *EGR1*, *JUN*, *COX5B*, *P2RY12*, *BDNF*, *SLC17A6*, *BAD*, *MFGE8*, *FOS*) relative to control cases. GO enrichment analysis revealed associations of these significantly dysregulated genes with pathways implicated in processes such as AP-1 complex signalling, pri-miRNA transcription, Smad-signalling, neuron projection and death, post-translational protein modification, and acute-phase response (Fig. 1B). Importantly, as the number of significantly dysregulated genes in this analysis is relatively low, the GO findings must be interpreted with caution. Thus, we also employed a competitive gene set analysis, CAMERA, which considers whole shifts in expression of groups of genes based on fold changes. Neuron development, projection and differentiation gene sets were downregulated in C9-ALS-FTSD cases relative to controls, as well as gene sets for synaptic structure, plasticity and transmission, cell projection organization and cytochrome c release; blood microparticle, platelet degranulation, endopeptidase inhibitor activity and inflammatory response gene sets were upregulated (Fig. 1C). Finally, microglia-specific genes were found to be significantly upregulated in our dataset, in line with our previous findings,^24^ further supporting an increase in microglial activation^24^ (Table 3).

**Table 3:** Cell type-specific dysregulation in C9-ALS-FTSD based on Brain RNA-Seq data

Gene set	No. genes	Direction	*P*-value	FDR
human_microglia_5_times	113	Up	8.58 × 10^−4^	0.03089637
human_microglia_10_times	78	Up	0.00983159	0.08848435
human_endothelial_top100	12	Up	0.02976191	0.13392859
human_microglia_top100	38	Up	0.03978398	0.15913592
human_endothelial_5_times	18	Up	0.04770685	0.17174467
human_endothelial_10_times	13	Up	0.05512601	0.18041241
human_fetal_astrocytes_5_times	46	Down	0.13938532	0.34892127
human_neuron_5_times	19	Down	0.21064688	0.42102263
human_fetal_astrocytes_10_times	22	Down	0.22756045	0.42102263
human_neuron_top100	5	Down	0.24559653	0.42102263
human_neuron_10_times	8	Down	0.27457446	0.44930366
human_fetal_astrocytes_top100	7	Down	0.67226571	0.84000835
human_mature_astrocytes_5_times	9	Up	0.67667339	0.84000835
human_oligodendrocyte_5_times	24	Down	0.73334664	0.86196644
human_mature_astrocytes_10_times	5	Up	0.76421218	0.86196644
human_mature_astrocytes_top100	7	Down	0.76619239	0.86196644
human_oligodendrocyte_top100	21	Up	0.80590044	0.8624924
human_oligodendrocyte_10_times	14	Up	0.81457615	0.8624924

FDR = false discovery rate.

Clustering of gene expression in C9-ALS-FTSD cases across the whole panel revealed the existence of two distinct gene expression signatures, herein referred to as neuroinflammatory panel signature 1 and 2 (NPS1 and NPS2). These signatures defined two disease subgroups and were delineated by the direction of expression of two gene clusters (Fig. 1D). The clearest phenotypic distinctions between NPS1 and NPS2 observed were that of manually graded glial TDP-43 burden and language impairment as determined by the ECAS, with highest TDP-43 burden and language impairment only occurring in NPS1. No clear segregation was observed for demographic (i.e. sex) or other phenotypic data (i.e. region of onset, disease duration). GO analysis of C9-ALS-FTSD gene clusters revealed an enrichment of immune and inflammatory response pathways in Gene Cluster 1, such as positive regulation of interleukin-8, NF-κB and interferon-γ responses (Fig. 1E). By contrast, Gene Cluster 2 exhibited an enrichment of axonal transport and synaptic signalling pathways (Fig. 1E). To determine which genes within the panel were contributing to the delineation of these clusters, differential expression analysis was conducted to identify differentially expressed genes between cases exhibiting NPS1 and NPS2 signatures. Forty-seven genes were included in a new clustered heat map (herein referred to as the NPS-defining gene list), exemplifying a clearer contrast between the direction of expression of genes between the two signatures (Fig. 1F). These genes were mostly from original Gene Cluster 2, related to axonal transport and synaptic signalling (Fig. 1G).

### Differentially expressed genes correlate with immunohistochemical staining features in C9-ALS-FTSD

**Figure 2:**
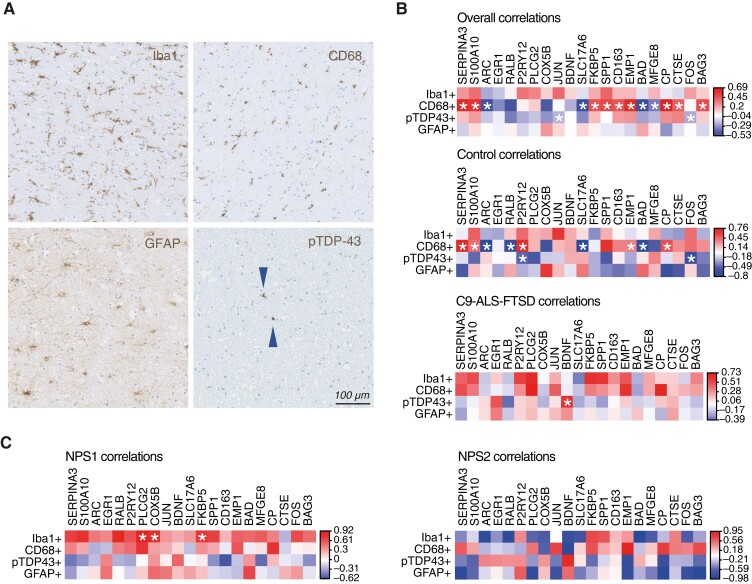
**Differentially expressed genes correlate with microglial and pTDP-43-related immunohistochemical features in C9-ALS-FTSD.** (**A**) Example images of immunohistochemical stains quantified using QuPath for correlations with NanoString neuroinflammation panel housekeeping-normalized counts. (**B**) Control-specific, C9-ALS-FTSD-specific, and overall significant correlations of prominence scores for Iba1, CD68, pTDP-43 and GFAP stains with NanoString normalized counts for 20 dysregulated genes in C9-ALS-FTSD from Fig. 1A. (**C**) NPS1- and NPS2-specific correlations of prominence scores for Iba1, CD68, pTDP-43 and GFAP stains with NanoString normalized counts for 20 dysregulated genes in C9-ALS-FTSD from Fig. 1A. Spearman’s *R* correlation coefficients are indicated by colour and correlations with a *P*-value < 0.05 are marked with an asterisk.

To explore the relationship of differentially expressed genes in C9-ALS-FTSD with glial activation and TDP-43 burden (Fig. 2A), we correlated microglia, astrocyte, and TDP-43-related immunohistochemical data from our previous digital pathology study^24^ with transcript counts of the 20 differentially expressed genes identified in Fig. 1. These data consisted of digitally extracted features (i.e. stain-positive superpixel counts, a measurement of stain abundance) from stained motor cortex (BA4) of the same C9-ALS-FTSD cohort included in the current study, and included Iba1 (i.e. homeostatic microglia), CD68 (i.e. activated macrophage), GFAP (i.e. activated astrocyte) and pTDP43 (i.e. phosphorylated TDP-43 aggregate) staining. Expression levels of several genes were found to correlate significantly with the number of CD68+ or pTDP43+ superpixels, with positive correlations between CD68+ and proinflammatory genes (e.g. FKBP5, CD163, SPP1), as well as with molecular chaperone regulator BAG3, and negative correlations between pTDP43 and the expression of JUN and FOS, subunits that form the transcription factor complex activator protein 1 (AP-1) (Fig. 2B). When subdivided by disease status, the significant proinflammatory gene expression correlations with CD68+ were lost in controls and a significant negative correlation of homeostatic microglia marker *P2RY12* expression with pTDP43+ appeared (Fig. 2B). Finally, a positive correlation with expression of the growth factor *BDNF* with pTDP43+ superpixels was seen in C9-ALS-FTSD but not controls (Fig. 2B). Interestingly, when cases were divided by NPS, NPS1 cases exhibited more positive correlations with stain abundance, while NPS2 correlation coefficients were more often negative (though non-significant). These data suggest distinct NPS-related directionality in correlations between gene expression and pathological features such as TDP-43 aggregation and glial activation (Fig. 2C), in line with our observation that C9-ALS-FTSD cases with a predominance of NPS1 gene expression tended to have higher glial TDP-43 aggregation burden (Fig. 1D).

### FKBP5 expression correlates significantly with clinical metric of executive dysfunction for C9-ALS-FTSD

**Figure 3:**
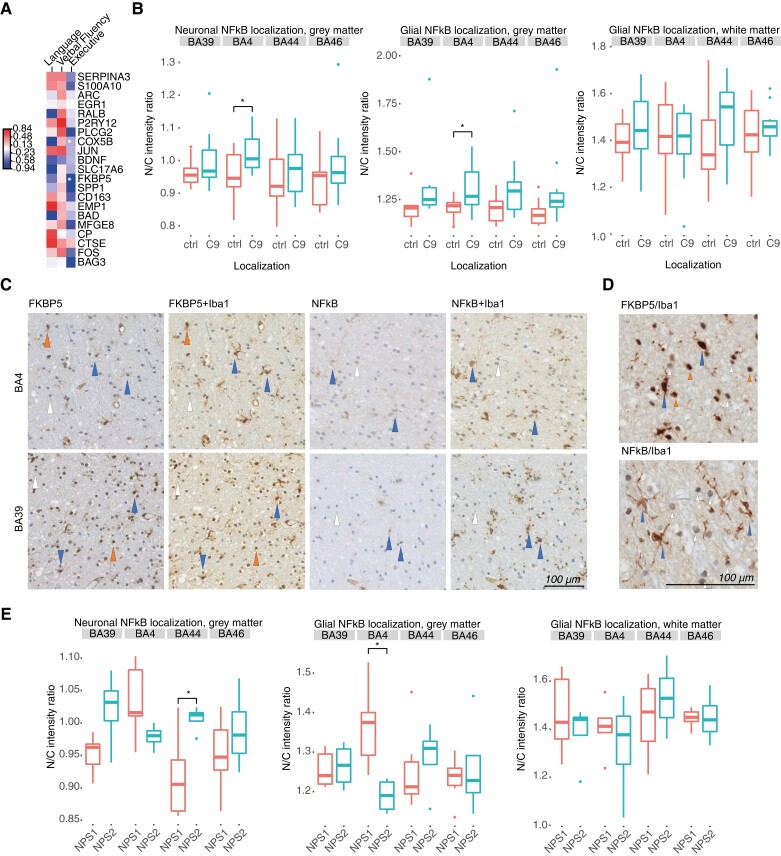
**FKBP5 expression correlates significantly with clinical metric of executive dysfunction**. (**A**) Correlation between language, fluency and executive scores from Edinburgh Cognitive and Behavioural ALS Screen (ECAS) and NanoString normalized counts for 20 dysregulated genes in C9-ALS-FTSD from Fig. 1A. Spearman’s *R* correlation coefficients are indicated by colour, and correlations with a *P*-value < 0.05 are marked with an asterisk. (**B**) Nuclear/cytoplasmic NF-κB intensity ratio quantification for neuronal (*left*), grey matter glial (*middle*) and white matter glial (*right*) staining between amyotrophic lateral sclerosis (ALS) and controls, stratified by brain region. *P < 0.05. (**C**) Double staining with FKBP5 or NF-κB and Iba1 to identify microglia-specific staining in BA4 (motor) and BA39 (extramotor; language). Microglia positive for FKBP5 or NF-*κ*B are indicated with blue arrows, other positive glia are indicated with orange arrows, and other negative glia are indicated with white arrows. (**D**) Increased magnification images of FKBP5 + Iba1 and NF-κB + Iba1 staining, with positive/negative glia indicated as described in **B**. (**E**) Nuclear/cytoplasmic NF-κB intensity ratio quantification for neuronal (*left*), grey matter glial (*middle*) and white matter glial (*right*) staining between NPS1 and NPS2, stratified by brain region. *P < 0.05.

To investigate possible relationships between differential transcription patterns and cognition, we examined correlations between differential gene expression and ECAS scores for the 20 genes we identified as most differentially expressed between C9-ALS-FTSD cases and controls (Fig. 3A). *FKBP5* and *COX5B* were found to negatively correlate with executive score. These relationships must be interpreted with caution as gene expression was measured in the motor cortex and not regional correlates of ECAS scores. However, it may be that changes in the motor cortex are reflective of changes in the relevant regions or a global cortical burden of disease. The immunophilin FK506-binding protein 51 (FKBP5) modulates inflammation through NF-κB signalling,^52,53^ and forms a chaperone complex with a heat shock protein (HSP90) in response to stress.^54^ We interrogated whether this was also the case in C9-ALS-FTSD brain tissue by using immunohistochemistry, rather than *in situ* hybidization, as the functional form of NF-κB is a protein whose cellular localization and expression level determines its function. Serial tissue sections were stained with FKBP5 and NF-κB and compared between control and C9-ALS-FTSD tissue. No evidence of a significant increase in nuclear/cytoplasmic FKBP5 intensity ratios was observed, though there was a general trend towards an increase in C9-ALS-FTSD (Supplementary Fig. 1). However, significant increases in nuclear/cytoplasmic NF-κB intensity ratios were found in BA4 grey matter in C9-ALS-FTSD, suggesting upregulation of this pathway in disease (Fig. 3B). Upon sequential staining of the same FKBP5- or NF-κB-stained tissue with Iba1, cell type-specific staining was observed for both FKBP5 and NF-κB (Fig. 3C). Notably, microglia were found to be FKBP5+, accompanied by both FKBP5+ and FKBP5− neuroglia of other subtypes. Contrastingly, microglia were the only glial subtype found to be NF-κB+ (Fig. 3D). Finally, when cases were stratified by inflammatory signature, NPS1 cases exhibited significantly higher nuclear/cytoplasmic NF-κB ratios in grey matter glia in BA4, and significantly lower ratios in neurons in extramotor BA44 (Fig. 3E).

### BDNF expression correlates significantly with disease duration in C9-ALS-FTSD

**Figure 4:**
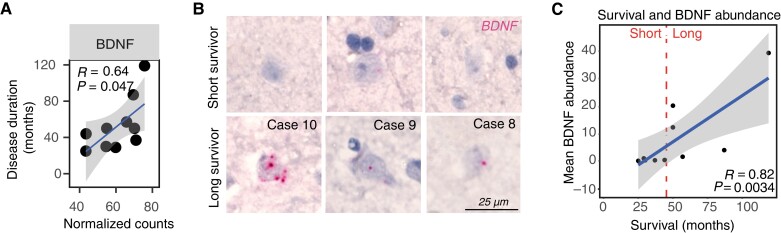
**BDNF expression correlates with disease duration in C9-ALS-FTSD.** (**A**) Significant correlation between brain-derived neurotrophic factor (*BDNF*) expression and disease duration. Spearman’s *R* and *P*-value is shown with a linear regression line and 95% confidence interval. (**B**) Example images of BaseScope™ *in situ* hybridization of *BDNF* probes in short and long survivors. (**C**) Significant correlation between mean *BDNF* transcript abundance (product score) and survival [i.e. disease duration (months)]. Spearman’s *R* and *P*-value is shown with a linear regression line and 95% confidence interval.

To explore relationships between expression of the 20 differentially expressed genes in C9-ALS-FTSD and disease progression, gene expression was correlated with disease duration and ALSFRS slope of decline, identifying a positive correlation between *BDNF* expression and disease duration (Spearman’s *R* = 0.64, *P =* 0.047) (Fig. 4A). BaseScope™ *in situ* hybridization, a highly sensitive method for quantification of transcript abundance,^55^ was used to validate our finding that *BDNF* expression correlates with disease duration in C9-ALS-FTSD. *BDNF* expression in BA4 was manually graded using a product score to account for both cell and regional transcript abundance. *BDNF* was predominantly expressed in neurons, with heterogeneous abundance, at both the cell and regional level (Fig. 4B). We confirmed a positive correlation between *BDNF* expression and disease duration (Fig. 4C). Individuals with a short disease duration (i.e. less than 48 months post-onset)^56^ consistently showed lower levels of *BDNF* expression, while individuals with long disease duration (i.e. more than 48 months post-onset) exhibited higher expression.

### Distinct inflammatory signatures exist across C9-ALS-FTSD, sporadic ALS and SOD1-ALS cohorts

**Figure 5:**
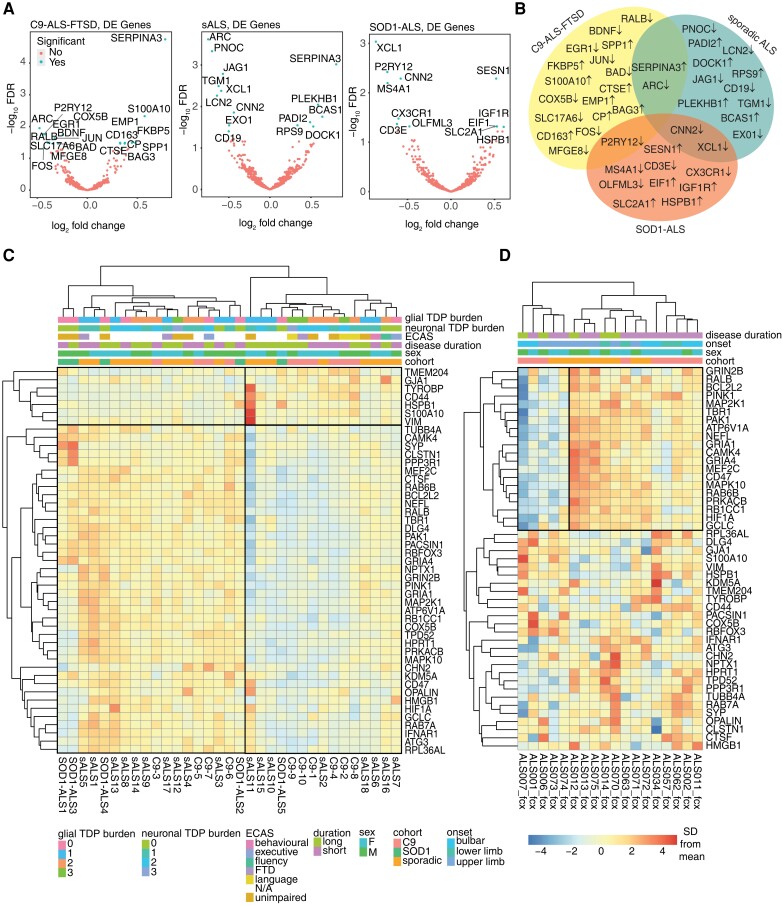
**C9-ALS-FTSD, sporadic ALS and SOD1-ALS cohorts exhibit both overlapping and unique neuroinflammatory signatures.** (**A**) Volcano plot showing differentially expressed (DE) genes for C9-ALS-FTSD (*n =* 10), sporadic ALS (sALS) (*n =* 18) or SOD1-ALS (*n* = 5) compared to controls by log_2_ fold change and −log_10_*P*-value. Non-significant genes are represented in red and significant genes above the Benjamini-Hochberg false discovery rate (FDR) threshold (i.e. *P*-adjusted < 0.05) are represented in blue. (**B**) Venn diagram showing the overlap of differentially expressed genes from **A** across cohorts, with arrow indicating direction of differential expression. (**C**) Clustered heat map with filtered neuroinflammatory panel signature (NPS) gene list from Fig. 1F, showing two distinct neuroinflammatory signatures across C9-ALS-FTSD, sALS and SOD1-ALS cohorts. Gene Clusters 1 and 2 are boxed, with opposite directions of expression. Demographic (cohort, sex), clinical (disease duration, ECAS) and pathological (pTDP-43 burden) keys are shown. (**D**) Clustered heat map with filtered NPS gene list from Fig. 1F, showing two distinct neuroinflammatory signatures in the frontal cortex across C9-ALS-FTSD and sALS cases from an independent, publicly available dataset, delineated particularly by the 20 genes comprising the top quadrants of the heat map. Key is the same as in **C**. ECAS = Edinburgh Cognitive and Behavioural ALS Screen; F = female; frontotemporal dementia; M = male; N/A = not applicable; SD = standard deviation.

To interrogate whether the observed NPS were specific to C9-ALS-FTSD or common across multiple ALS cohorts, we next applied the nCounter neuroinflammation panel to sALS (*n* = 18) and SOD1-ALS (*n* = 5) cohorts (Table 2). Differentially expressed genes between each cohort and controls were largely different, with no overlap of genes passing the FDR threshold present in all three cohorts (Fig. 5A and B). GO analysis of these genes revealed both distinct and shared significant terms across cohorts related to immune function and proteostasis, as well as other pathways (Supplementary material). Distinct terms included response to interleukin, microRNA gene transcription, neuronal death, post-translational protein modification, aggrephagy and chaperone-mediated protein transport in C9-ALS-FTSD; cell development and morphogenesis in sALS; and translation initiation, protein kinase B signalling, chaperone-mediated protein folding in SOD1-ALS. Overlap included postsynaptic neurotransmission in C9-ALS-FTSD and sALS, glial migration in C9-ALS-FTSD and SOD1-ALS, and chemokine-mediated signalling, and T cell, B cell and natural killer cell processes in sALS and SOD1-ALS (Supplementary material). Despite these differences, heat map cluster analysis of C9-ALS-FTSD, sALS and SOD1-ALS cases using the filtered NPS gene list revealed two distinct NPS subgroups, present across the included cohorts, again delineated by the expression of two gene clusters related to immune response or axonal transport and synaptic processes (Fig. 5C and Supplementary Fig. 2A and B for full panel and GO). The two subgroups also did not appear to segregate clearly based on our available clinical metrics for cognitive function or glial pTDP-43 burden, unlike what was observed within the C9-ALS-FTSD cohort alone. However, it is worth noting that pTDP-43 is a marker of cytoplasmic aggregation, which may not necessarily reflect loss of TDP-43 function or early aggregation events; future studies looking at the emergence of cryptic exons or other functional measures of TDP-43 pathology in bulk RNA sequencing datasets are warranted to examine this further. Interestingly, differential expression analysis revealed significant dysregulation of immune response genes (i.e. complement and microglial genes) in cognitively impaired cases (C9-ALS-FTSD, sALS) (Supplementary Fig. 2C and D) while only one significantly dysregulated gene, *CNN2*, was detected between unimpaired cases (C9-ALS-FTSD, sALS) and controls. As such, it is possible that cognitively impaired cases have convergent disease mechanisms despite being from different cohorts, while unimpaired cases may be too diverse to detect significant dysregulation in this context.

To test the generalizability of the filtered NPS gene list from C9-ALS-FTSD cases across ALS cohorts, the list was next applied to the clustering analysis including cases from all cohorts (Fig. 5C). Genes clustered in the same way as in the C9-ALS-FTSD analysis (Fig. 1E) and groups were delineated such that C9-ALS-FTSD cases remained divided as previously. Opposite directions of expression were most apparent in the 40 genes encompassed within the clades comprising the lower quadrants of the heat map (Fig. 5C). The gene list was next tested further on an independent cohort of post-mortem frontal cortex and cerebellum of eight C9-ALS and 10 sALS cases from publicly available RNA sequencing data^26^ (Fig. 5D). Strikingly, in the frontal cortex, clustering analysis revealed a very distinct delineation between NPS1 and NPS2, present across both cohorts and particularly defined by the direction of expression of 20 of 47 of the genes in the filtered list (Fig. 5D). Notably, this effect did not persist in the cerebellum, for which the clustering analysis using the filtered gene list did not reveal distinct subgroups (Supplementary Fig. 2E), highlighting the possibility of region-specific signatures.

## Discussion

This study investigated neuroinflammatory differences in deeply clinically phenotyped ALS post-mortem tissue, allowing us to compare molecular data directly with motor and cognitive as well as immunohistochemical features. We found an upregulation of microglia-specific genes in C9-ALS-FTSD, substantiating previous findings of microglial dysregulation in cases with this genetic background.^17,24^ Microglia have been shown to require C9orf72 for normal function in *C9orf72*^−/−^ microglia and peripheral myeloid cell models, demonstrating a pro-inflammatory response as a result of C9orf72 knockout.^22,23^ Thus, haploinsufficiency resulting from *C9orf72* HRE may lead to the microglial dysregulation observed both in this study and elsewhere.

Two genes whose expression was found to correlate with clinical scores (*BDNF*, *FKBP5*) were further validated with spatial resolution using immunohistochemistry or BaseScope™ *in situ* hybridization. C9-ALS-FTSD-related increased nuclear localization, and thus activation of FKBP5 signalling partner, NF-κB, was observed in neurons and glia in both motor and extramotor regions, along with exhibition of microglia-specific NF-κB staining in white matter. Activation of NF-κB is associated with the release of pro-inflammatory cytokines, such as TNF-α and IL-1β,^57^ which have been shown cause neurotoxicity in various contexts.^58,59^ It is indicative of an upregulation of an inflammatory response mediated by IKKα/β kinases,^60^ which are negatively regulated by autophagy.^61,62^ It is possible that the more pro-inflammatory signature, the increased BA4 grey matter glial NF-κB activation, and the tendency toward higher TDP-43 burden seen in C9-ALS-FTSD cases with NPS1 is related to lower levels of negative regulation via autophagy. Indeed, the *NLRP3* inflammasome, as well as several autophagy-related genes (i.e. *ATGs*), are part of Gene Cluster 1; activation of *NLRP3* is also increased with autophagy deficiency.^61,63,64^ Thus, therapeutic studies involving the use of immunomodulatory or autophagy-targeting drugs may seek to consider stratification of cases based on molecular signatures of inflammation to ensure the meaningful measurement of outcomes. Additionally, such trials may also benefit from monitoring NF-κB activation to assess target engagement and therapeutic efficacy; activation status could be obtained by profiling levels of pro-inflammatory cytokines associated with NF-κB signalling in participant blood samples. Further, as NF-κB activation in tissue-resident mononuclear cells (i.e. microglia) has been observed in the present study and in other recent works, circulating peripheral blood mononuclear cell (PBMC) transcriptomes could provide a proxy measure of such activation.^61,65^

The utility of transcriptome data for identifying molecular signatures that correlate with survival has been recently demonstrated, identifying a subgroup of ALS patients with poorer survival and differential expression of genes related to glial signalling.^66,67^ Here we identify *BDNF* expression as a clinical correlate of survival, highlighting the additional mechanistic insights afforded through our targeted approach. Expression of *BDNF* in the motor cortex was found to be downregulated in disease, and positively correlated with disease duration, suggesting a protective effect. BDNF signalling has been previously demonstrated to have either neuroprotective^68^ or indirectly excitotoxic effects.^69^*BDNF* expression has also been shown to correlate with decreased cognition^70^. Importantly, many preclinical studies investigating the effects of BDNF in ALS are biased towards SOD1 mouse models,^71,72^ in which increased BDNF-TrkB is observed.^73^ Further, a phase III clinical trial conducted using recombinant methionyl human BDNF did not demonstrate therapeutic benefit, though this study and further trials conducted thereafter did not stratify genetically; importantly, *C9orf72* mutations in ALS had not been discovered at the time.^74,75^ In contrast to SOD1 models, this study shows that *BDNF* expression is downregulated in C9-ALS-FTSD post-mortem tissue, and C9-ALS-FTSD cases appear to have a more inflammatory background; thus, BDNF-related treatments may function differently in a C9-ALS-FTSD context. Expression of *BDNF* by immune cells was found to promote neuronal survival in human tissue culture.^76^ Moreover, subcutaneous perfusions of BDNF have been shown to reverse microglial activation in aged mice,^77^ perhaps through indirect downregulation of microglial MHC-II expression.^78^ As such, cell type-specific manipulation of *BDNF* expression may provide a more nuanced approach to controlling microglial activation and neuronal loss. In addition to a possible treatment, BDNF could also have utility as a biomarker for disease prognosis. Recently, BDNF and pro-BDNF levels in CSF were shown to be associated with survival in ALS patients; in line with our discussion, C9-ALS patients showed significantly lower serum BDNF levels than non-carriers.^79^ Finally, monitoring of BDNF levels to assess both therapeutic efficacy and participant safety, particularly given its hormetic nature and the potential for excitotoxicity, will be important in trials investigating modulators of BDNF signalling, such as TrkB receptor agonists.^80^

While other recent approaches have used RNA sequencing to investigate and identify important gene expression signatures across the transcriptome,^66,67,81,82^ our targeted approach to investigating neuroinflammatory signatures without amplification bias ensured a focused evaluation of neuroinflammation specifically. We identified two distinct molecular profiles, NPS1 and NPS2, with immune response terms enriched in Gene Cluster 1 and axon transport and synaptic signalling terms enriched in Gene Cluster 2. These signatures do not segregate clearly with known demographic, clinical or pathological data across cohorts suggesting that these signatures are not readily identifiable through visible features. These signatures are present in multiple ALS cohorts within our study and in an independent publicly available dataset, underscoring their generalizability and, crucially, highlighting the importance of molecular stratification in clinical trials. Clinical trials may benefit from employing stratification methods based on molecular markers rather than, or in addition to, genetic and clinical criteria, as without stratification a positive effect of treatments on a particular subgroup may be obscured. For example, the recent macrophage-targeted sodium chlorite trial (NP001) showed no overall effect on the primary outcome measure.^83^ However, subsequent subgroup analysis showed that those that did have a beneficial therapeutic response to the drug had higher than average levels of circulating IL-18 and LPS (akin to our NPS1), implying that molecular stratification by key circulating inflammatory markers could enable us to treat a subset of ALS patients for whom inflammation plays a more substantial role.^83^ Our data would suggest that a combinatorial blood-based biomarker approach,^84^ using circulating markers derived from a gene panel such as ours and validated across distinct ALS patient populations (as in Fig. 5D), would be a more appropriate way to identify subgroups that would benefit from targeted therapies. Promising candidates are based on the 20 genes from our NPS-defining gene list that strongly delineate clusters in an independent, publicly available dataset (i.e. *GRIN2B*, *RALB*, *BCL2L2*, *PINK1*, *MAP2K1*, *TBR1*, *PAK1*, *ATP6V1A*, *NEFL*, *GRIA1*, *CAMK4*, *GRIA4*, *MEF2C*, *CD47*, *MAPK10*, *RAB6B*, *PRKACB*, *RB1CC1*, *HOF1A*, *GCLC*) and two additional NPS-defining genes (*CD44* and *TYROBP*) that also appear in a recently identified gene list defining a molecular subgroup relating to glial activation.^67^ Molecular stratification, in the form of tissue-derived and circulating biomarkers, is the mainstay of patient stratification for clinical trials in oncology^85^; given the convergence of these studies with our data, molecular subtyping should be considered for future trials implementing targeted therapies in people with ALS.

## Supplementary Material

awad243_Supplementary_Data

## Data Availability

The datasets supporting the conclusions of this article are included in this published article and its Supplementary material, or available in the figshare repository. SD numbers of cases from the Edinburgh Brain Bank included in the study are available upon request.
