# Supplementary material for: Distinct neuroinflammatory signatures exist across genetic and sporadic amyotrophic lateral sclerosis cohorts
Source: Brain. 2023 Jul 14;146(12):5124–38. doi: 10.1093/brain/awad243 (PMC10690026; doi:10.1093/brain/awad243)
Supplement: awad243_Supplementary_Data [file awad243_supplementary_data.pdf]

Supplement: Distinct neuroinflammatory signatures exist across genetic and sporadic ALS cohorts by Rifai et al.

Olivia M. Rifai,[1,2,3,4,5,*] Judi O'Shaughnessy,[2,4] Owen R. Dando,[3,5,6] Alison F. Munro,[7] Michael D. E. Sewell,[1,3] Sharon Abrahams,[8] Fergal M. Waldron,[9] Christopher R. Sibley4,5,6,10 and Jenna M. Gregory[9]

1 Translational Neuroscience PhD Programme, Centre for Clinical Brain Sciences, University of Edinburgh, Edinburgh, EH8 9XD, UK

2 Centre for Clinical Brain Sciences, University of Edinburgh, Edinburgh, EH16 4SB, UK

3 UK Dementia Research Institute, University of Edinburgh, Edinburgh, EH16 4SB, UK

4 Euan MacDonald Centre for Motor Neurone Disease Research, University of Edinburgh, Edinburgh, EH16 4SB, UK

5 Centre for Discovery Brain Sciences, University of Edinburgh, Edinburgh, EH8 9XD, UK

6 Simons Initiative for the Developing Brain, University of Edinburgh, Edinburgh, EH8 9XF, UK

7 Cancer Research UK Edinburgh Centre, Institute of Genetics and Cancer, The University of Edinburgh, Edinburgh, EH4 2XU, UK

8 Human Cognitive Neuroscience-Psychology, School of Philosophy, Psychology and Language Sciences, University of Edinburgh, Edinburgh, EH8 9AD, UK

9 Institute of Medical Sciences, University of Aberdeen, Aberdeen, AB25 2ZD, UK

10 Institute of Quantitative Biology, Biochemistry and Biotechnology, School of Biological Sciences, University of Edinburgh, The King's Buildings, Edinburgh, EH9 3FF, UK

*Correspondence to: OM Rifai, Centre for Discovery Brain Sciences, 15 George Square, Edinburgh, UK EH8 9XD. E-mail: olivia.rifai@ed.ac.uk

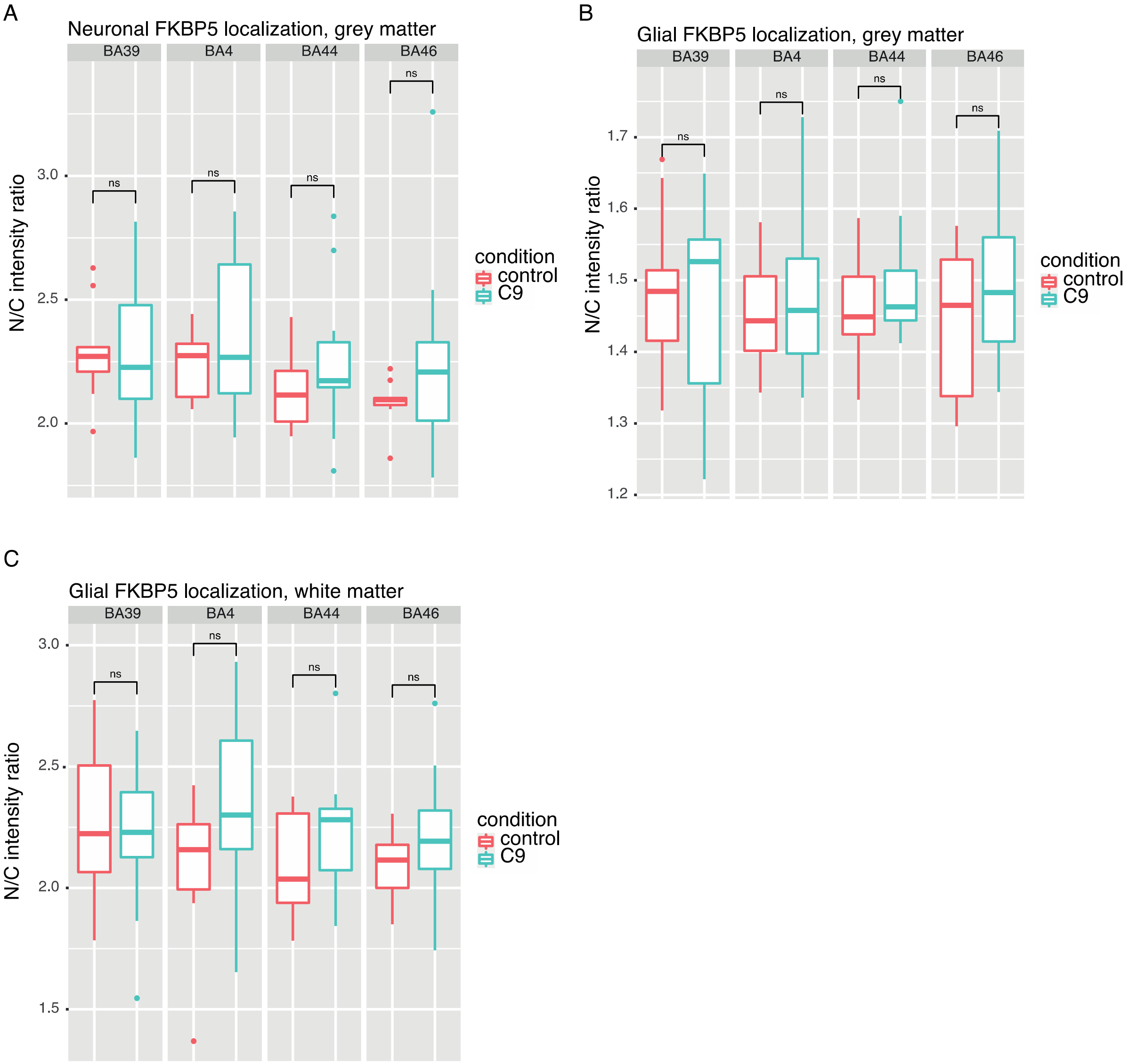

Supplementary Fig. 1. Supplementary FKBP5 analyses.

Nuclear/cytoplasmic FKBP5 intensity ratio quantification for neuronal, grey matter glial, and white matter glial staining between C9-ALS-FTSD and controls, stratified by brain region. * $p < 0.05$.

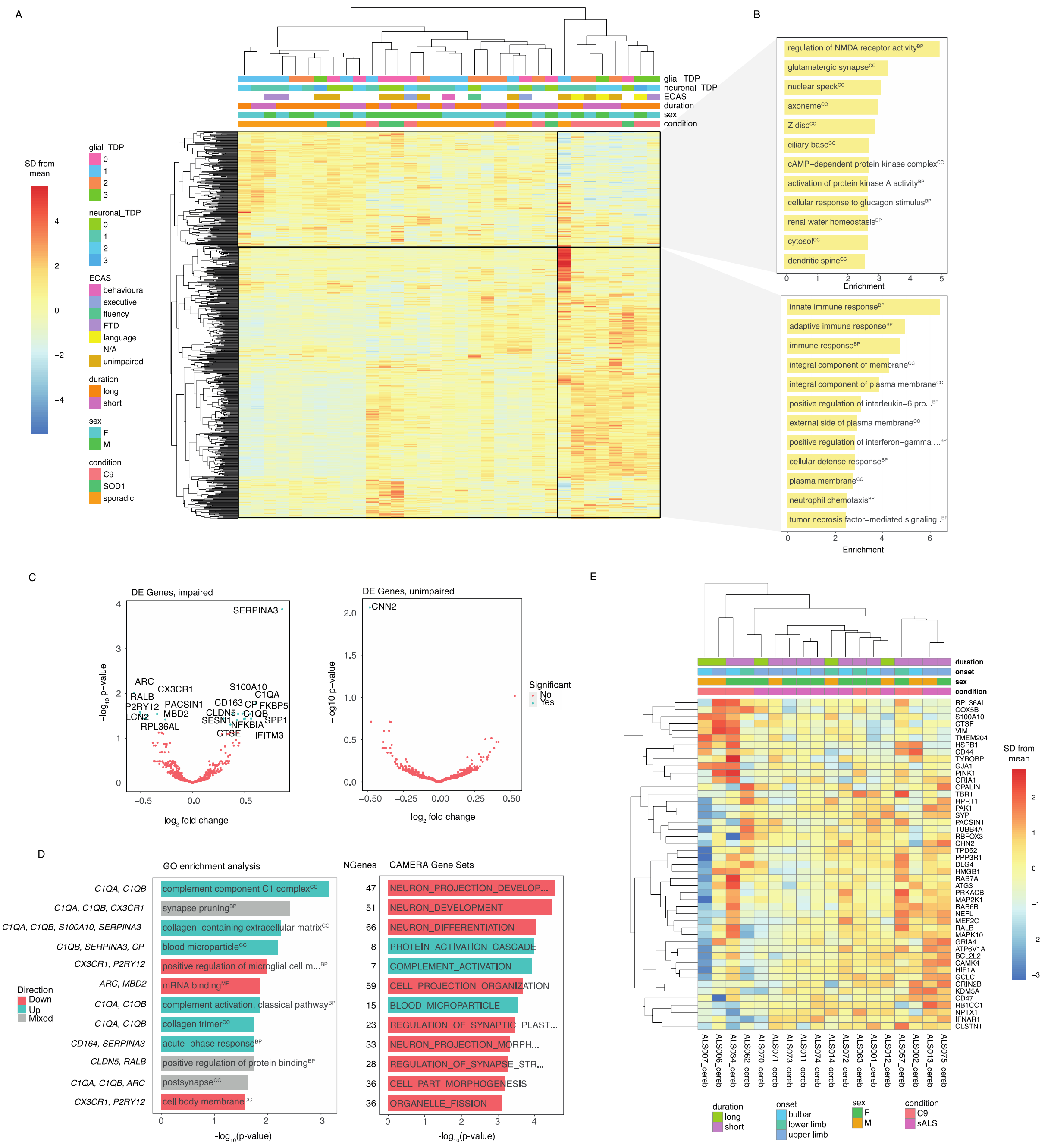

Supplementary Fig. 2. Supplementary heatmap analyses.

(a) Clustered heatmap with entire neuroinflammation panel (770 genes), showing two distinct neuroinflammatory signatures across C9-ALS-FTSD, sALS, and SOD1-ALS cohorts. Demographic (cohort, sex), clinical (disease duration, ECAS) and pathological (pTDP-43 burden) keys are shown. (b) GO enrichment analysis for gene clusters that define signatures 1 and 2 showing top 12 dysregulated gene sets. Italicised terms are downregulated. (c) Volcano plot showing differentially expressed genes for cognitively impaired (C9-ALS-FTSD and sALS) or cognitively unimpaired (C9-ALS-FTSD and sALS) cases compared to controls by $\log_2$ fold change and -$\log_{10}$ FDR. (d) (left) GO enrichment analysis of genes enriched in cognitively impaired cases by type with -$\log_{10}$(p-value) score showing top 12 dysregulated gene sets; MF, molecular factor; CC, cellular component; BP, biological process. Italicised terms are downregulated; key genes for each term are shown to the left; (right) CAMERA gene set analysis of gene sets enriched in cognitively impaired ALS cases showing top 12 dysregulated gene sets, with the number of genes for each term shown to the left. (e) Clustered heatmap with filtered NPS gene list from Fig. 1F, showing the expression of neuroinflammatory genes in the cerebellum across C9-ALS and sALS cases from an independent, publicly available dataset, delineated particularly by the first 20 genes.

Online supplementary data on figshare.com

Rifai, Olivia (2023): 1. Digital pathology analysis scripts. figshare. Online resource. https://doi.org/10.6084/m9.figshare.21916557.v1

Rifai, Olivia (2023): 2. Raw images from digital pathology analysis. figshare. Figure. https://doi.org/10.6084/m9.figshare.21916575.v2

Rifai, Olivia (2023): 3. Raw and normalised NanoString counts. figshare. Dataset. https://doi.org/10.6084/m9.figshare.21916581.v1

Rifai, Olivia (2023): 4. Differential expression analysis results. figshare. Dataset. https://doi.org/10.6084/m9.figshare.21916629.v1

Rifai, Olivia (2023): 5. GO enrichment and GSA results. figshare. Dataset. https://doi.org/10.6084/m9.figshare.21916656.v1

Rifai, Olivia (2023): 6. C9-ALS gene clusters list. figshare. Dataset. https://doi.org/10.6084/m9.figshare.21916671.v1
